# Imaging properties of 3D printed breast phantoms for lesion localization and Core needle biopsy training

**DOI:** 10.1186/s41205-020-00058-5

**Published:** 2020-02-18

**Authors:** Arafat Ali, Rifat Wahab, Jimmy Huynh, Nicole Wake, Mary Mahoney

**Affiliations:** 1 Department of Radiology, University of Cincinnati Medical Center, 234 Goodman Street, Cincinnati, OH 45267 USA; 2 Department of Radiology, Montefiore Medical Center, 111 East 210th Street, Bronx, NY 10467 USA; 3 Department of Radiology, NYU Langone Health, Center for Advanced Imaging Innovation and Research (CAI2R) and Bernard and Irene Schwartz Center for Biomedical Imaging, New York, 10016 NY USA

**Keywords:** 3D printing, Core needle biopsy, Breast biopsy, Breast phantom

## Abstract

**Background:**

Breast cancer is the most commonly diagnosed malignancy in females and frequently requires core needle biopsy (CNB) to guide management. Adequate training resources for CNB suffer tremendous limitations in reusability, accurate simulation of breast tissue, and cost. The relatively recent advent of 3D printing offers an alternative for the development of breast phantoms for training purposes. However, the feasibility of this technology for the purpose of ultrasound (US) guided breast intervention has not been thoroughly studied.

**Methods:**

We designed three breast phantom models that were printed in multiple resins available through Stratasys, including VeroClear, TangoPlus and Tissue Matrix. We also constructed several traditional breast phantoms using chicken breast and Knox gelatin for comparison. These phantoms were compared side-by-side for ultrasound penetrance, simulation of breast tissue integrity, anatomic accuracy, reusability, and cost.

**Results:**

3D printed breast phantoms were more anatomically accurate models than traditional breast phantoms. The chicken breast phantom provided acceptable US beam penetration and material hardness for simulation of human breast tissue integrity**.** Sonographic image quality of the chicken breast phantom was the most accurate overall. The gelatin-based phantom also had acceptable US beam penetration and image quality; however, this material was too soft and poorly simulated breast tissue integrity. 3D printed phantoms were not visible under US.

**Conclusions:**

There is a large unmet need for a printable material that is truly compatible with multimodality imaging for breast and other soft tissue intervention. Further research is warranted to create a realistic, reusable and affordable material to 3D print phantoms for US-guided intervention training.

## Background

Breast cancer is the most commonly diagnosed cancer in women (24.2%) and the leading cause of cancer related deaths worldwide (15%) [1]. Diagnostic mammograms with adjunctive ultrasound (US) are proven to aide in tumor detection and improve diagnostic accuracy, with the ultimate aim of early cancer diagnosis and reducing unnecessary biopsies [2]. Ultrasound guided core needle biopsy (CNB) is an indispensable tool for breast cancer diagnosis, particularly in BIRADS 4 and 5 breast lesions. The high diagnostic accuracy of ultrasound guided CNB makes it the procedure of choice for the pathological diagnosis of breast abnormalities identified on US, which can reduce and occasionally obviate the need for surgical management [3].

CNB is frequently performed by breast fellowship and non-fellowship trained radiologists. Therefore, improving resident competency during the formative years of training is crucial. Phantom based procedural training is a highly successful methodology for improving user confidence and mitigating patient risk [4–6]. Hands-on training is particularly important for US-guided procedures, which require coordination of both the imaging probe and procedural instrument.

Currently, there are limited options for US lesion localization and US-guided procedural training. Commercially biopsy phantoms are available at a cost of ranging from $350–450 each. However, this option is cost prohibitive for most institutions given their limited reusability. Consequently, the majority of training institutions utilize food or animal product phantoms to mimic soft tissue [7–10]. These types of phantoms have several limitations, ranging from sanitary concerns to accurate tissue simulation both visual and tactile. Based on our anecdotal experience after multiple biopsy training seminars, the ideal phantom would be anatomically accurate, allow adequate visualization of target lesions, emulate breast tissue integrity, retain its structure for multiple uses, maintain cleanliness of the equipment, and be affordable.

The relatively recent advent of medical 3D printing has opened a new avenue for the accurate production of models of the human body for medical education [11, 12]. In the context of breast imaging, several papers have explored the benefit of 3D printed phantoms for image quality analysis, surgical planning, and implantable bioprinted breast scaffolds [13–16]. Researchers have also created phantoms from 3D printed molds filled with ultrasound compatible polymers [17, 18]. However, to our knowledge, no 3D printed phantoms which accurately mimic breast tissue characteristics, are feasible for multimodality imaging, and are produced at a low cost, exist. In this observational study, we compared 3D printed breast phantoms with traditional breast phantoms (food/animal product phantoms), using multiple commercially available materials. The 3D printed breast phantom models were evaluated for their sonographic imaging quality and feasibility for US-guided biopsy training, with the intent of producing a low-cost reusable 3D breast model.

## Materials and methods

Three different 3D printed breast phantoms were custom designed for the purpose of imaging evaluation and biopsy training [Table 1]. The first was model (phantom 1) was printed using a rigid resin combination of VeroClear and VeroBlue (Connex500, Stratasys, Rehovot, Israel). A similar design method was used for phantom 2, which was subsequently printed with the softer TangoPlus resin. A third model (phantom 3) was created from magnetic resonance imaging data. The fat and fibroglandular tissue (FGT) were segmented and converted to an STL file format for printing. To best mimic human tissue properties, the fat was printed with a combination of A30Clear coated at 600 μm around Tissue Matrix and the FGT was printed with a ShoreA50 combination of VeroClear and A30Clear (J750, Stratasys, Rehovot, Israel).

**Table 1:** Breast Phantom quality assessment

	*Phantom Material*	*Pros*	*Cons*	Material Hardness*
*Phantom 1*	VeroClear and VeroBlue	Sanitary Anatomically accurate	No US penetrance Poor tissue integrity simulation Expensive Not reusable	Shore D = 83–86
*Phantom 2*	Tango Plus	Sanitary Anatomically accurate	No US penetrance Poor tissue integrity simulation Expensive Not reusable	Shore A = 26–28
*Phantom 3*	Fat: Tissue Matrix and A30Clear FGT: VeroClear and A30Clear	Sanitary Realistic tissue integrity simulation Anatomically accurate	No US penetrance Expensive Not reusable	Shore A = 30
*Phantom 4*	Chicken Breast with pimento olive targets	Excellent ultrasound penetration with easily visible target lesions Realistic tissue integrity simulation Affordable	Unsanitary Not reusable Anatomically inaccurate	Shore-000 = 36
*Phantom 5*	Knox Gelatin with blueberry targets	Excellent ultrasound penetration with easily visible target lesions Affordable	Unsanitary Excessively soft integrity Not reusable Anatomically inaccurate	Shore-00 = 10

^*^Hardness values provided by manufacturer or based on established values of comparable materials [20, 21].

Traditional ultrasound procedural phantoms were constructed using both chicken breast (phantom 4) and gelatin (phantom 5). The gelatin-based breast phantom was created from Knox gelatin (Associated Brands, Medina, NY), using methods cited by previous authors [8, 19]. For this simulation, we used an approximately 16 oz. model without added preservatives. Pimento olives were placed in the chicken phantom and blueberries were placed in the gelatin-based phantom to simulate breast masses.

Ultrasound imaging was performed with a Philips Equip 5G (Bothell, Washington) using a linear 5–18 MHz and 5–12 MHz transducer, as well as a Siemens Acuson 3000 (Malvern, Pennsylvania) with a 18 L6 HD 5.5–18 MHz transducer.

Each breast phantom was evaluated for ultrasound penetration, simulation of breast tissue integrity, anatomic accuracy, reusability, and cost. A fellowship trained breast radiologist (RW) assessed the qualitative sonographic and tactile comparison of each model to human breast tissue. This anecdotal comparison is summarized with a semiquantitative evaluation of each model using a Likert scale [Table 2]. A rating of 1 was the lowest possible score and a rating of 5 was the best possible score for each category. For ultrasound beam penetration, a score of 1 was given for no penetration, 2–4 was given for progressively increased beam penetration and a score of 5 was given for complete beam penetration. For anatomic accuracy, scoring was based on resemblance of the phantom to a human breast. A score of 1 was given for no anatomic resemblance, 2–4 was given for varying degrees of anatomic resemblance, and a score of 5 was given for complete anatomic resemblance. For reusability, a score of 1 was given if the model is unusable for percutaneous biopsies, a score of 2 for a single biopsy training seminar, a score of 3 or 4 for three to ten biopsy training seminars, and a score of 5 for greater than ten biopsy training seminars. Each biopsy training seminar consists of a minimum of four trainees attempting several biopsies through the phantom.

**Table 2:** Breast phantom likert assessment

	*Phantom Material*	*Ultrasound Beam Penetration*	Anatomic Accuracy	*Reusability*
*Phantom 1*	VeroClear and VeroBlue	1	5	1
*Phantom 2*	Tango Plus	1	5	1
*Phantom 3*	Fat: Tissue Matrix and A30Clear FGT: VeroClear and A30Clear	1	5	1
*Phantom 4*	Chicken Breast with pimento olive targets	5	1	2
*Phantom 5*	Knox Gelatin with blueberry targets	5	1	2

Material hardness values were referenced from established values for all materials except gelatin [20, 21]. Gelatin hardness is dependent upon the type and concentration of ingredients utilized; therefore, the reference value provides only an estimate of similar hardness gelatin products.

## Results

**Fig. 1:**
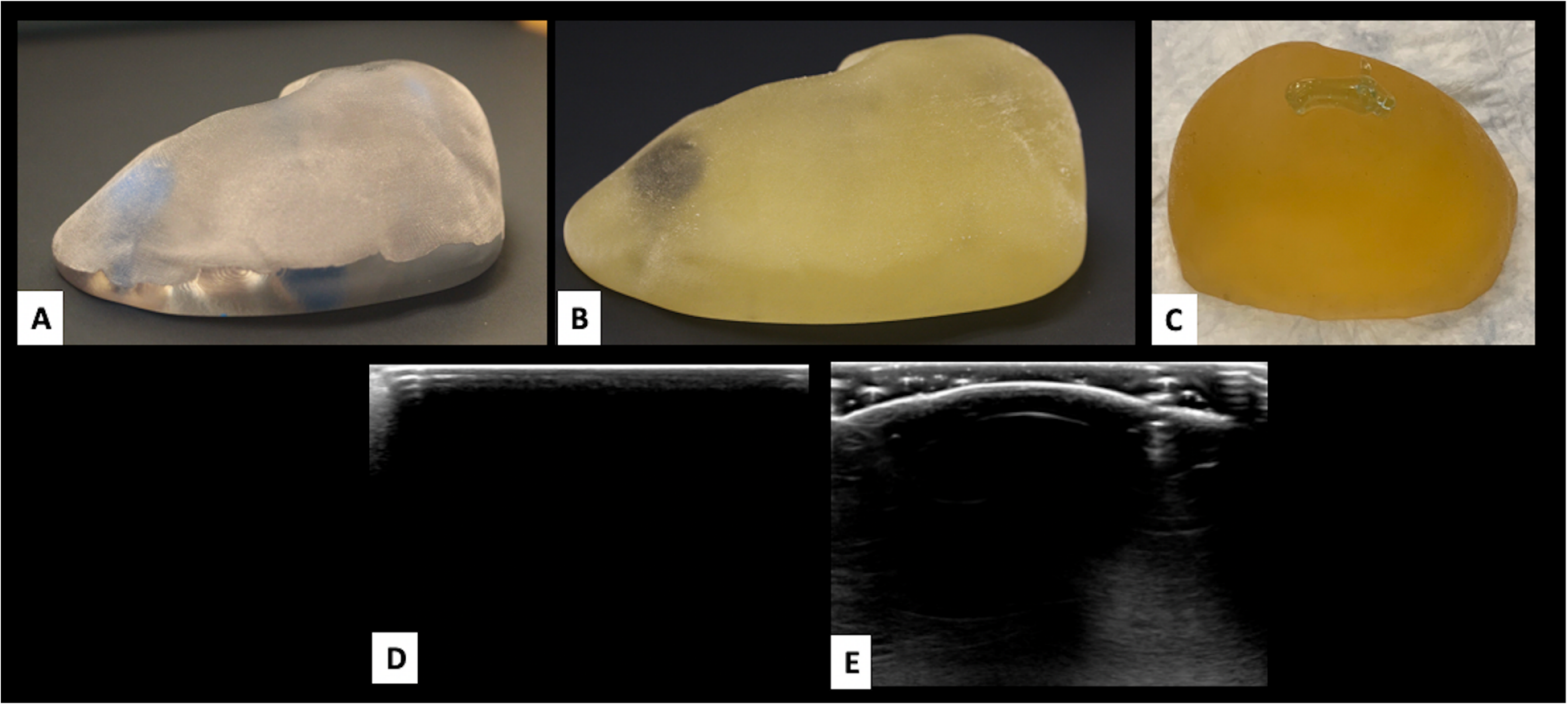
3D printed Breast Phantoms. Phantoms were printed in VeroClear (**a**), TangoPlus (**b**) and a new combination of TissueMatrix with a coating of VeroClear at 600 μm (**c**). Ultrasound (US) image (**d**) of the TangoPlus phantom demonstrates lack of sound penetration. Images obtained with standoff pad (**e**) shows similar findings. Both VeroClear and TissueMatrix phantoms had similar results on US imaging

3D printed breast phantoms were more anatomically accurate models compared to traditional breast phantoms. TissueMatrix material was the most accurate in terms of tactile simulation of breast tissue. VeroClear and TangoPlus were too rigid compared to normal breast tissue. The most pronounced limitation of the 3D printed phantoms was the lack of US beam penetration [Fig. 1]. Various adjustments in imaging parameters, including the use of a standoff pad were unsuccessful in creating an acceptable image for CNB training purposes.

**Fig. 2:**
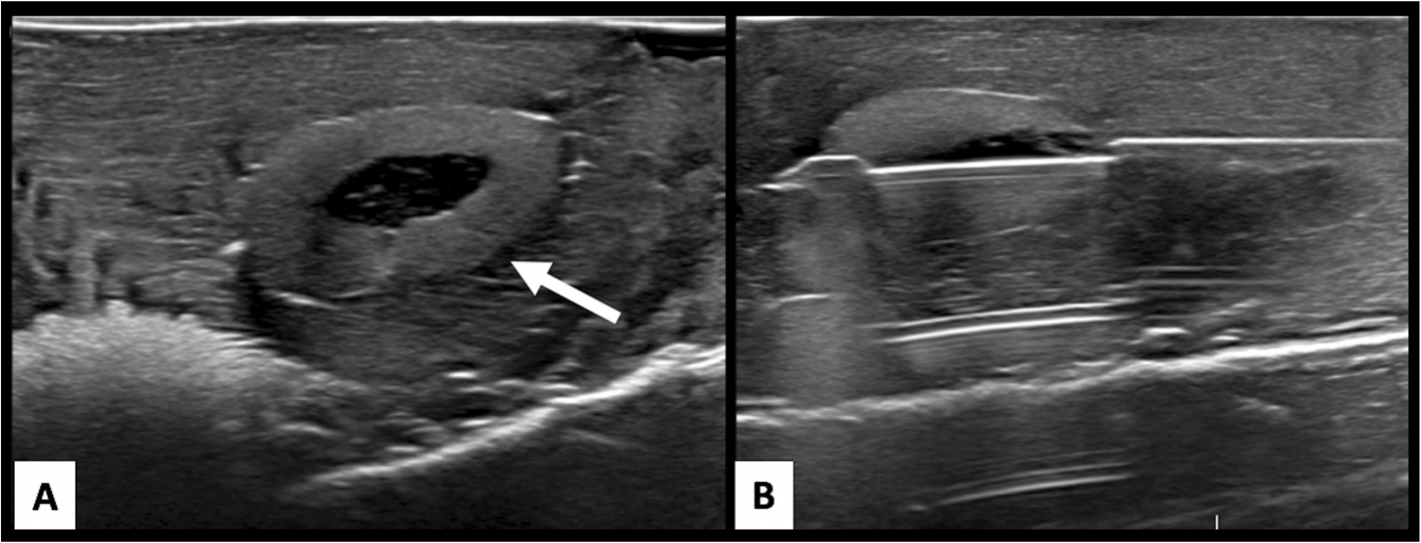
Chicken breast phantom biopsy simulation. US images of a chicken breast (**a**) phantom with an embedded pimento olive (arrow) to simulate breast lesion. Images from “lesion” biopsy (**b**) taken with a Celero 12-gauge device show near accurate imaging appearance compared with true ultrasound guided breast biopsy

**Fig. 3:**
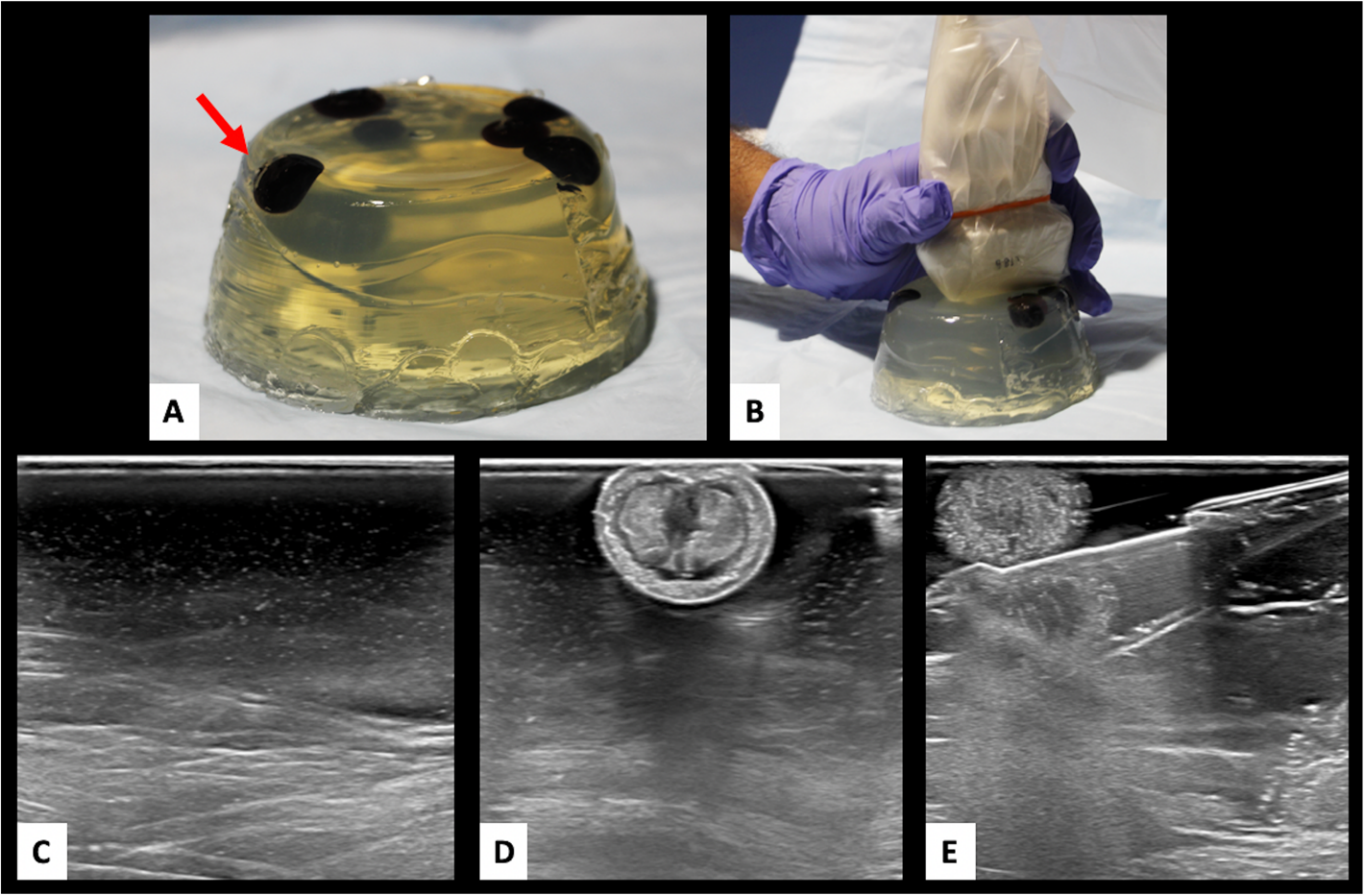
Knox gelatin phantom biopsy simulation. Knox gelatin breast phantom (**a** and **b**) phantom with embedded blueberries (red arrow) to simulate breast lesion was tested for ultrasound image quality. Ultrasound images (**c** and **d**) demonstrate adequate ultrasound penetration and target lesion visibility. However, images with Celero 12-gauge device (**e)** are slightly obscured by air related artifact from the biopsy device

**Fig. 4:**
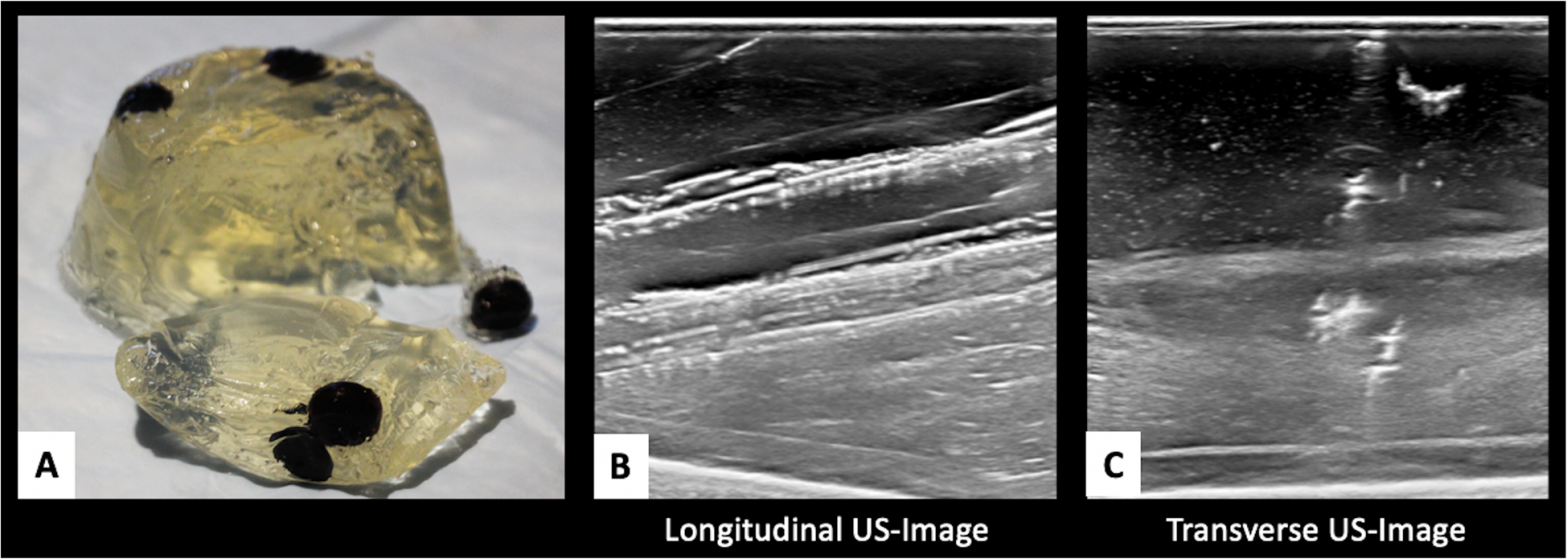
Fragility of Knox gelatin phantom. Knox gelatin breast phantom (**a**) easily fragments with excessive pressure. Ultrasound images after several biopsy attempts create linear air tracts (**b** and **c**), reducing visibility of target lesions

The chicken breast phantom fared the best overall with acceptable US beam penetration, image quality and material hardness for simulation of human breast tissue integrity**.** Sonographic image quality of the chicken breast phantom was also the most accurate overall [Fig. 2]. The gelatin-based phantom had acceptable US beam penetration and image quality, however the tactile simulation quality compared with human breast tissue was poor [Fig. 3]. Gelatin proved too soft, allowing the biopsy needle to glide too easily through the phantom. Furthermore, repeated biopsies create air tracks within the gelatin phantom and obscured visualization of target lesions [Fig. 4].

## Discussion

To master US-guided CNB, a radiologist must fluidly move the biopsy device accurately through the breast, while imaging the target with the contralateral hand. Hands-on training with breast phantoms is necessary to hone these skills. Several commercial ultrasound training phantoms exist, but are costly and have limited reusability.

3D printed breast models have been used to in preoperative planning, simulation of multiple surgical techniques, and as phantoms for assessing imaging parameters for both MRI and x-ray based mammography [13–15, 22–26]. Conceptually, 3D printed models are also feasible for US-guided procedural training, provided the printed material is adequate for proprioceptive simulation of real human tissue and image production.

The need for printable soft tissue density material has been well addressed. New materials including TangoPlus, and more recently Tissue Matrix, provide a realistic tissue feel. However, the sonographic image quality of these materials is not well studied. The ultrasound beam was unable to penetrate all three of the printed breast model materials including VeroClear, TangoPlus, and Tissue Matrix. Various US imaging parameters were utilized to optimize the image, including a gel standoff pad, without success in producing a diagnostic quality image. The reasoning for poor image quality may be multifold and is not completely understood. The printing process likely results in deposition of air between the resin layers, which makes sound waves impenetrable. Among the 3D printed materials, Tissue Matrix came the nearest to realistic breast soft tissue simulation. The remaining materials were too hard for a needle to penetrate, making them poor models for CNB training.

At our institution, a chicken breast phantom with implanted pimento olives has been the most realistic teaching tool. Sonographic visualization of soft tissues and simulated breast lesions was excellent. The chicken breast reasonably simulated the proprioceptive characteristics of human breast biopsy. However, several drawbacks of this method still exist. Foremost, the chicken breast method suffers from environmental unfriendliness and risks contamination of imaging equipment. For this reason, biopsy instruments used on the chicken model are often disposed of after each session, leading to additional waste and increased cost.

US phantoms created from Knox brand gelatin provide an alternative avenue for low-cost biopsy training. Several authors have used this product for breast as well as other US-guided procedural training [8, 18]. Similar to chicken breast, the sonographic image quality of gelatin models adequately simulates human soft tissue. However, limitations exist with the gelatin-based phantom. Gelatin is particularly fragile and the density too soft compared to human breast tissue. Small cracks created in the gelatin during routine handling, multiple biopsies, or excessive pressure from the probe can create pockets of air within the model and obstruct visualization of targets. The short shelf life of the gelatin and creation of air tracks with each biopsy limits reusability. Preservatives may be added to extend shelf life; however, the single use nature of gelatin models makes preservatives unnecessary.

Limitations of this study include the lack of additional types of printing technologies or materials, including flexible materials printed with the use of material extrusion, vat photopolymerization, and other types of material jetting printers (i.e. 3D Systems and Mimaki).

## Conclusion

There is a large unmet need for printable materials that are truly compatible with the multimodality imaging necessary for breast and other soft tissue intervention. Although CT compatible materials are well studied, research on the sonographic properties of these materials is lacking. Further research is warranted to create a realistic, reusable and affordable material to 3D print phantoms for ultrasound-guided intervention training.

## Data Availability

The datasets used and/or analyzed during the current study are available from the corresponding author on reasonable request.

## Abbreviations

CNB: Core needle biopsy
US: Ultrasound
